# Resilience evaluation of low-carbon supply chain based on improved matter-element extension model

**DOI:** 10.1371/journal.pone.0301390

**Published:** 2024-04-01

**Authors:** Xiaochun Luo, Kai Kang, Lin Lu, Changliang Yu, Chaoling Li, Beibei Li, Song Hu, Xia Qi, Yaomei Zhou

**Affiliations:** 1 School of Economics and Management, Nanjing University of Aeronautics and Astronautics, Nanjing, China; 2 School of Economics and Management, Guangxi Normal University, Guilin, China; 3 School of Safety Science and Emergency Management, Wuhan University of Technology, Wuhan, China; 4 Jose Rizal University, Metro Manila, Philippines; Industrial University of Ho Chi Minh City, VIET NAM

## Abstract

How to evaluate the resilience level and change trend of supply chain is an important research direction in current supply chain management practice. This paper proposes a new method of supply chain resilience assessment based on hesitant fuzzy linguistic term set (HFLTS) and matter element extension theory. Firstly, based on the research status quo at home and abroad, a low-carbon enterprise supply chain resilience assessment index system is established, which includes six first-level indicators and corresponding 21 second-level indicators of product supply resilience, resource resilience, partner resilience, information response resilience, financial resilience and knowledge resilience. Secondly, HFLTS was used to collect expert opinions and Ordered Weighted Arithmetic (OWA) to calculate the expert composite language, by which the fuzzy evaluation matrix of supply chain resilience assessment indicators was obtained. Once again, the resilience indicator weights are determined based on a game-theoretic portfolio assignment method combining the best-worst method (BWM) and the CRITIC method. Finally, the nearness degree function is combined with the extension comprehensive evaluation method to improve the matter element extension model, and the supply chain resilience assessment model of low-carbon enterprises based on the game theory combination assignment-improved matter element extension is established. Taking X low-carbon enterprise as an example, the evaluation results show that the supply chain resilience level of this enterprise is II, and the eigenvalue of the grade variable is 2.69, and the supply chain resilience is shifting to III, and the supply chain resilience is shifting to III, which indicates that the supply chain resilience of this enterprise is being enhanced. Therefore, the improved matter element extension not only ensures the accuracy of the evaluation results, but also has higher prediction accuracy.

## 1. Introduction

Low-carbon supply chain is the intrinsic core of green economy and circular economy, and it is an important focus point for promoting high-quality economic development, which has been widely concerned by the industry and society [1]. There-fore it is necessary to create a low-carbon supply chain system to promote the development of low-carbon enterprises [2]. However, in recent years, as the risk of global uncertainty increases, unexpected events such as trade protectionism and geopolitical conflicts occur frequently [3]. Enterprises face a range of problems in today’s market environment, such as reduced demand, insufficient supply of raw materials, production constraints, backlogs of finished products, and logistical disruptions [4]. In particular, for low-carbon companies, the scale and concentration of their operations have led to poor supply chain resilience [5]. Once a supply chain is exposed to a large external shock, it may lead to the consequences of supply chain disruption, which will affect the continued normal operation of the supply chain [6].

Supply chain disruptions are characterised by uncertainty, unpredictability and disruption [7]. When an uncertain event occurs, the products in the supply chain cannot flow normally, which makes the low-carbon enterprises in the supply chain face severe operational and financial risks, and ultimately affects the performance of the supply chain [8]. A resilient low-carbon supply chain can not only predict the risk of disruption quickly, but also quickly take relevant measures to quickly recover from the disruption, thus reducing the impact of supply chain disruption [9]. Therefore, it is of great significance to scientifically assess the resilience level of low-carbon supply chains and enhance supply chain resilience based on the assessment results for the long-term development of low-carbon enterprises.

Scholars have conducted extensive research on the evaluation methods of supply chain resilience, and although there are many evaluation methods of resilience nowadays, most of them evaluate resilience through a single language, ignoring the influence of the uncertainty of the real situation on the decision-making opinions of managers. Therefore, the composite linguistic expression based on hesitant fuzzy linguistic term set (HFLTS) proposed in this paper not only provides a richer expression than the single linguistic value, but also this composite linguistic expression is based on the real situation, which is also more flexible. And the results calculated using the OWA operator can simplify the calculation process based on HFLTS. In addition, this study also combines the game theoretic combinatorial empowerment and the improved matter element topologisable model to explore the supply chain resilience evaluation model in a relevant way. The purpose of this thesis is to answer the following two questions:

RQ1: In the evaluation system of supply chain resilience, how to fully respond to the opinions of relevant experts and reasonably allocate the weights of resilience indicators?
RQ2: How to judge the level of supply chain resilience and the trend of change?

The following are the prospective innovations of this study: firstly, considering the complexity of supply chain resilience index data in the real situation, this paper innovatively introduces the composite language of HFLTS and OWA computational experts, which reduces the ambiguity of information. Moreover, the game theory combination assignment method reduces the influence of subjective and objective factors on the assignment of indicators. Secondly, this paper introduces the nearness degree function to improve the matter element extension model, and constructs a new supply chain resilience dynamic early warning model, which provides a reference for the relevant managers of low-carbon enterprises to construct the supply chain resilience dynamic early warning system.

## 2. Literature review

### 2.1. Supply chain resilience

In the concept of supply chain management, supply chain resilience is a systemic adaptive capability that enables a supply chain to plan quickly when faced with uncertain risks and maintain normal operations by resolving them [10, 11]. Therefore, supply chain resilience can be considered not only as a dynamic capability, but also as a concept that covers the different phases of a supply chain from the onset of a risk to its recovery: preparing, responding, recovering, and growing or adapting [12, 13], and that a resilient supply chain not only predicts and analyses the risks in advance, but also is able to quickly return to the initial state after a disruption in the supply chain or even achieve a better than before operation state [14].

Some scholars have conducted theoretical research on improving supply chain resilience according to the different stages of supply chain risk occurrence, and put forward many valuable supply chain resilience ideas. Dey [15] believes that in the preparation stage, the supply chain can monitor and warn supply chain risk through digital technology to identify the risk in a timely manner, improve the prediction ability of the supply chain, and then improve the resilience of the supply chain. Ipek et al. [16] argued that when supply chain risk occurs, the supply chain’s rapid response capability can be improved by improving the flexibility and agility of the supply chain to avoid the risk of supply chain disruption and maintain the normal operation of the supply chain. According to Ivanov et al. [17], supply chain resilience can be improved by enhancing supply chain flexibility, redundancy and collaboration among partners at a later stage after supply chain risks occur. In addition, Mohammed et al. [18] and Kumar [19] argue that upstream and downstream firms in a supply chain can improve the original supply chain structure and increase the level of supply chain resilience through shared learning, absorptive capacity, and innovative capabilities.

It can be seen that strengthening one aspect of the supply chain may not have a significant effect on improving the overall resilience strength of the supply chain. This is due to the fact that the supply chain is a dynamic system connecting different organisations, and therefore presents a complex network structure, where firms in the supply chain interact and depend on each other, which means that supply chain resilience is affected by the collaborative relationship between firms, intimacy, supply chain complexity, and supply chain length [20, 21]. Therefore, once some nodes in the supply chain have problems, it may cause the devastating impact of supply chain failure. In response to this unfavourable situation, enterprises should consider improving supply chain resilience as an important strategic decision and the resilience element as an important indicator for supply chains to maintain competitive advantage [22, 23].

### 2.2. Current situation of supply chain resilience assessment

The assessment of supply chain resilience strength is the fundamental basis for supply chain system optimisation and enhancement, and academic assessment methods for supply chain resilience can be divided into deterministic and fuzzy language according to language. In terms of deterministic language, Kaviani et al. [24] proposed a method to measure supply chain resilience based on grey system theory approach. They identified distribution problems and supply constraints as the most serious vulnerabilities that threaten the normal operation of supply chains. Qi et al. [25] used the introduction of buffer operator to improve the grey prediction model and combined it with the approximation of the ideal solution ranking method (TOPSIS) firstly to predict the dynamic prediction of supply chain resilience level. Mohammed et al. [26] used the hierarchical analysis method (AHP) to compute supply chain resilience indicator weights, and use TOPSIS to rank the product suppliers with resilience, according to the evaluation results obtained can provide useful help for managers to improve the resilience of the supply chain. Xu et al. [27] established a resilience evaluation model for the supply chain of material security based on the matter element extension model, and confirmed the feasibility of the model, which provides valuable reference standards for the decision maker’s management.

In terms of fuzzy language, Mohammed et al. [28] introduced fuzzy mathematics into the hierarchical analysis method and constructed a supply chain network resilience evaluation model based on the AHP-TOPSIS method, using AHP to calculate the weights of the indicators, and applying the TOPSIS method to judge fuzzy multi-objective planning models. Ekanayake et al. [29] used a fuzzy comprehensive evaluation method, and from the supply chain vulnerability perspective and constructed a multilevel multi-criteria supply chain resilience evaluation model. Wang et al. [30] evaluated the green building supply chain resilience based on the network analysis method—fuzzy integrated evaluation method and gave suggestions for improvement based on the evaluation results. Gu et al. [31] proposed an integrated method, which combines the optimal worst-case method and the fuzzy TODIM method, to analyse the port congestion, labour shortage and abnormal freight rate fluctuation scenarios to improve the resilience of the maritime supply chain.

In summary, although many scholars in the field of supply chain resilience have proposed methods for supply chain resilience evaluation, there are still some problems: first, most of the existing studies construct supply chain resilience evaluation system from a single aspect of supply chain resilience, and the selected resilience indexes are not only not flexible enough, but also do not take into account that the resilience indexes in the real environment are vulnerable to the impacts of non-conventional factors, and ignore the widespread incompatibility problems in reality. Secondly, the existing supply chain resilience evaluation method cannot be used to evaluate the supply chain resilience. Secondly, the existing supply chain resilience evaluation methods cannot systematically predict the change trend of supply chain resilience, and it is difficult to realise dynamic resilience early warning.

**Fig 1:**
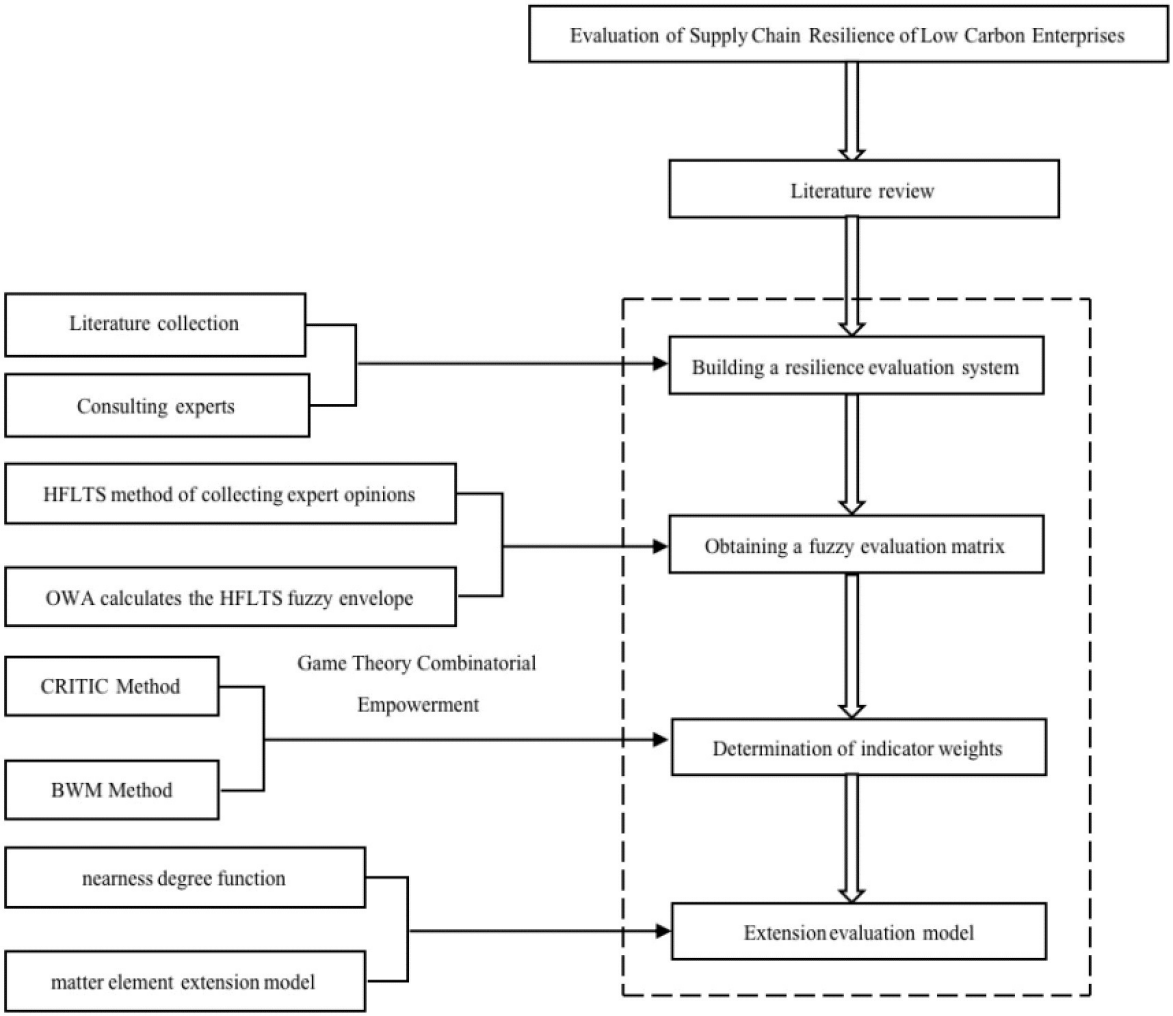
Evaluation process of supply chain resilience based on BWM-CRITIC-improved matter element extension model.

In order to fill the gaps in the above research, this paper proposes to construct a supply chain resilience evaluation model combining OWA, CRITIC-BWM and the improved matter element extension model. The OWA is introduced by the expert group to evaluate the indicators using the composite language and calculate the HFLTS fuzzy envelope of each language, and then quantify the evaluation matrix according to the weights of each expert; based on the CRITIC-BWM, we calculate the subjective and objective combination of the comprehensive weights of each indicator; Finally, we improve the matter element extension model by introducing the nearness degree function and determine the resilience level of each indicator, which can provide suggestions for supply chain risk prevention in a targeted way. The specific process is shown in Fig 1.

## 3. Supply chain resilience evaluation index system

Low-carbon supply chains are not only an important way to realise a low-carbon economy and reduce carbon emissions, but also a key to promoting the level of green development of supply chains. For low-carbon enterprises, improving supply chain resilience not only reduces the risk of supply chain disruption, but also promotes the sustainable development of low-carbon supply chains. On the basis of literature review and expert consultation, this paper expands and extends the resilience indexes for the characteristics of dynamic operation of supply chain of low-carbon enterprises, and constructs the evaluation system of supply chain resilience of low-carbon enterprises.

Firstly, the evaluation indexes are screened out by using literature frequency statistics, and the search interface of China Knowledge Network (CNN) and other databases are searched by inputting "supply chain resilience", "resilience", "resilience assessment", and so on. In the search interface of China Knowledge Network and other databases, inputting "supply chain resilience", "resilience", "supply chain performance", etc. to search, a total of 1,365 pieces of related literature were retrieved from 2015–2024, and 128 pieces of effective literature were finally selected by filtering and reviewing the evaluation, supply chain performance, supply chain operation and other related contents related to resilience; After reading the relevant literature, find out the factors that affect the proper operation of the supply chain and consult the relevant experts. ultimately, from the resilience of product supply, resource resilience, partner resilience, information response resilience, financial resilience, Finally, from the six aspects of product supply resilience, resource resilience, partner resilience, information response resilience, financial resilience, knowledge resilience, we establish the first-level indicators, and based on the connotation differences of the six first-level indicators, we finally select 21 second-level indicators as shown in Table 1.

**Table 1:** Evaluation system of supply chain resilience indicators of low carbon enterprises.

Dimension	primary indicators	Secondary indicators	Nature of indicators
Product supply resilience A_1_	Product supply scope B_1_	The quantity of the product category c_1_	quantitative
		Meet individual needs c_2_	qualitative
	Product availability time B_2_	Customer demand response time c_3_	quantitative
		Deliver Resilience c_4_	quantitative
	Product quantity resilience B_3_	Adapt to changes in the number of customer needs c_5_	qualitative
	Product supply efficiency B_4_	Order miss rate c_6_	quantitative
		Order delay rate c_7_	quantitative
		Order early delivery rate c_8_	quantitative
Resource resilience A_2_	Human resource security B_5_	Multiskilled workforce c_9_	qualitative
		Employee behavior flexibility c_10_	qualitative
		Employee Risk Awareness c_11_	qualitative
	Procurement Guarantee B_6_	Diversified procurement c_12_	qualitative
		Backup supplier c_13_	qualitative
		Contractual recourse option c_14_	qualitative
		Supplier reliability c_15_	qualitative
	Production equipment guarantee B_7_	Equipment reliability c_16_	quantitative
		Capacity redundancy c_17_	quantitative
	Logistics support B_8_	Inventory surplus c_18_	qualitative
		Optional shipping channel c_19_	qualitative
		Logistics company reputation c_20_	qualitative
Partner resilience A_3_	Cooperation closeness B_9_	Long term partnership with c_21_	qualitative
		Shared risk and shared benefits c_22_	qualitative
		Joint Decision c_23_	qualitative
	Information sharing degree B_10_	Sharing information c_24_	qualitative
		Trust each other c_25_	qualitative
	Synergistic partner B_11_	Change the time spent by the synergistic partner c_26_	qualitative
		Change the cost of synergy partners c_27_	qualitative
		Number of synergistic partners c_28_	quantitative
Information Response Resilience A_4_	Information Technology Level B_12_	IT infrastructure c_29_	qualitative
		Big Data Technology c_30_	qualitative
	Information responsiveness B_13_	Information response speed c_31_	quantitative
		Information response range c_32_	quantitative
	Information distribution accuracy B_14_	Supply chain visibility c_33_	quantitative
		Information distribution mechanism c_34_	quantitative
		Management Attention c_35_	qualitative
Financial resilience A_5_	Financing capacity B_15_	Supply Chain Finance c_36_	quantitative
		Total assets c_37_	qualitative
		Shareholding structure c_38_	qualitative
		Creditworthiness c_39_	qualitative
	Profitability B_16_	Gross margin c_40_	quantitative
		Sales revenue growth rate is c_41_	quantitative
		Inventory turnover days c_42_	quantitative
	Price resilience B_17_	Cost control c_43_	quantitative
		Price advantage c_44_	quantitative
	Market position B_18_	Market share c_45_	quantitative
		Brand awareness c_46_	qualitative
Knowledge resilience A_6_	Learning Organization B_19_	Learn from experience c_47_	qualitative
		Knowledge Sharing Culture c_48_	qualitative
		Interorganizational exchange of experiences c_49_	quantitative
	Collaborative Innovation B_20_	Collaborative technology innovation c_50_	qualitative
		Collaborative Management Innovation c_51_	qualitative
	Product Development B_21_	The cost required to develop a new product c_52_	quantitative
		The time required to develop a new product c_53_	quantitative

Product supply resilience is a measure of an enterprise’s ability to provide relevant products that consumers need in a timely manner [32]. Product supply resilience includes four secondary indicators: product supply scope, product supply time, product quantity resilience and product supply efficiency. The scope of product supply reflects the variety and quantity of products offered by the company and its ability to meet the individual needs of consumers. Product availability time includes customer demand response time and delivery resilience. Product quantity resilience reflects the degree to which the results of enterprises’ forecasts of consumer demand differ from the actual demand of consumers. Product supply efficiency reflects the efficiency of a company in supplying products within a time frame acceptable to customers.

Resource resilience includes human resource security, procurement security, production equipment guarantee and logistics guarantee. Human resource security reflects the awareness of relevant risks and the ability of human resources to have a variety of skills that can be adapted to different working environments and work requirements [33]. Procurement assurance reflects that when purchasing raw materials, enterprises have multiple backup suppliers, which can spread procurement risks to multiple suppliers and regions [34]. Production equipment guarantee reflects the ability of production equipment to ensure normal production when producing products [35]. Logistics guarantee is the ability of supply chain transportation channels to quickly and effectively adjust with changes in market demand to meet sudden needs [36, 37]. Generally speaking, the higher the human resource security, procurement guarantee, production equipment guarantee and logistics guarantee of low carbon enterprises, the higher the resilience level of the supply chain.

Partner resilience is reflected in the fact that enterprises in the supply chain can strengthen the collaboration of the supply chain through effective communication, and then be able to replace and adjust the capabilities of collaborating partners in a timely manner [38]. Partner resilience includes closeness of cooperation, degree of information sharing, and collaborative partners. Closeness of cooperation reflects the ability of companies in the supply chain to establish partnerships to address supply chain risks when faced with them [39, 40]. The degree of information sharing is the degree to which companies in the supply chain trust each other and share information [41]. Synergistic partners reflect not only the number of collaborators in the supply chain, but also the time and cost required to change the time it takes to change collaborators [42]. In addition, enterprises in the supply chain can also enhance supply chain collaboration through effective communication, trust and information sharing, thereby enhancing the speed of response to supply chain risks and ultimately improving supply chain resilience.

Information response resilience is the ability of enterprises in the supply chain to receive and process information. Information response resilience includes information technology level, information response ability and information distribution accuracy [43]. The level of information technology covers the development of digital technologies such as the Internet of Things, big data, cloud computing, machine learning, blockchain and cyberphysical systems of the entire supply chain, through which information technology can not only enhance the visibility and transparency of the supply chain, but also monitor and warn risks in the supply chain in a timely manner [44, 45]. Information responsiveness is the ability of enterprises in the supply chain to quickly adjust their production and operation activities based on external information [46]. Information distribution accuracy is the ability of supply chain information systems to distinguish between right and wrong information. Therefore, in order to improve the resilience of the supply chain, it is necessary to enhance the information response ability of enterprises.

Financial resilience includes financing capacity, profitability, price resilience, and market position. Financing resilience reflects the ability of enterprises in the supply chain to finance through their own credit level and total assets [47]. Profitability resilience reflects how profitable companies in the supply chain are to sell related products over a period of time [48]. Price resilience reflects the level of cost control and associated price advantages when a company achieves profitability [49]. Market position reflects the brand awareness of a company’s products in the market and the associated market share [50].

Knowledge resilience includes learning organizations, collaborative innovation, and product development. A learning organization is one in which an enterprise or organization can learn from experience, strengthen communication between organizations, share knowledge and culture between organizations, and make up for shortcomings after experiencing supply chain disruptions [51]. Collaborative innovation means that enterprises in the supply chain can find partners, integrate their resources and technologies, and realize collaborative technology innovation and management innovation [52]. Product development refers to the time and cost required by a company to develop a new product after it has an insight into a business opportunity [53]. By strengthening the learning organization, collaborative innovation ability and product development capability of the supply chain, it can not only promote communication between all members of the supply chain, alleviate the "bullwhip effect", but also enhance the core competitiveness of the supply chain and improve the resilience of the supply chain.

## 4. Research methods

### 4.1. HFLTS and OWA calculation of fuzzy envelope

As a most direct way to express opinions, language is often used as a single language in previous expert evaluations. However, for some complex indicator decision-making problems, the use of comparative language can more accurately express the expert’s opinion than a single language. Fuzzy language was proposed by scholar Zadeh, who advocated the use of linguistic variables as expressions of expert decision-making information as a way to reduce the complexity of expert language [54]. Subsequently, Rodriguez et al. [55] proposed the concept of hesitant fuzzy linguistic sets and they argued that decision makers may not always be able to accurately express information about evaluation metrics, and therefore HFLTS can be used to express decision makers’ hesitant preferences. Chiclana et al. [56], on the other hand, used OWA operators to compute the fuzzy language as a way of representing linguistic variables. Since hesitation fuzzy language has greater advantages for describing the evaluation information of decision makers, it is now used in various fields.

Therefore, in this paper, hesitant fuzzy language is employed to represent expert opinions. Therefore, this paper applies an algorithm that uses both single language and comparative language, in which there are three types of comparative language expressions, namely, fuzzy envelopes based on comparative language "at least *S*_*i*_" fuzzy envelopes based on comparative language "up to *s*_*j*_", and fuzzy envelopes based on comparative language "between *S*_*i*_ and *s*_*j*_ ". In this paper, the linguistic terminology set is organised according to the degree of influence of each indicator as: *S* = {*S*_0_,*S*_1_,*S*_2_,*S*_3_,*S*_4_} = {Weak, Weaker, Medium, Stronger, Strong}, $s_{0}^{*}=0$, $s_{1}^{*}=0.25$, $s_{2}^{*}=0.5$, $s_{3}^{*}=0.75$, $s_{4}^{*}=1$.

Considering that the authority of different experts in the field is different, different weights can be assigned to the evaluation of each expert, in which the expert is assigned the weight *w*_*t*_ = (*w*_*t*1_, *w*_*t*2_, ⋯,*w*_*tn*_)^*T*^ (n is the number of experts). In turn, the HFLTS fuzzy envelope can be calculated by OWA and weighted fuzzy matrix can be obtained with expert weights, and finally the evaluation matrix X can be obtained by the trapezoidal fuzzy number expectation expression. with the desired expression:

$$\bar{A}=(a+b+c+d)/4$$ (1)

### 4.2. Determination of the weight of evaluation indicators

#### 4.2.1. CRITIC method objective weighting

The CRITIC method belongs to a kind of objective assignment method, which is measured by the intensity of comparison and the conflictability between evaluation indicators, and the intensity of comparison can be expressed in the form of standard deviation. The greater the standard deviation and the conflictability between the factors, the higher the weight of the indicator; The specific steps are as follows:

$$A=\left[\begin{array}{cccc} a_{\text{1}1} & a_{12} & \cdots & a_{1n}\\ \vdots & \vdots & \vdots & \vdots \\ a_{m1} & a_{m2} & \cdots & a_{mn} \end{array}\right]$$ (2)

The A matrix represents a supply chain resilience evaluation system with m evaluation objects and n evaluation indicators.

In determining the supply chain resilience assessment indicators, the negative indicators are transformed positively for ease of calculation.

$$b_{ij}=\frac{\text{1}}{\lambda +\text{max}\left|a_{i}\right|+a_{ij}}$$ (3)

Where: ${b^{\prime }}_{ij}=\left\{\frac{b_{ij}-\text{min}(b_{i})}{\text{max}(b_{i})-\text{min}(b_{i})}\right.$ is the corresponding indicator value; *b*_*ij*_ is the homotropic indicator value; max|*a*_*i*_| indicates the maximum value of the ith indicator; The λ value is 0.1, indicating the coordination coefficient. After the above processing, the evaluation matrix *b* after forward can be obtained.


$${b^{\prime }}_{ij}=\left\{\frac{b_{ij}-\text{min}(b_{i})}{\text{max}(b_{i})-\text{min}(b_{i})}\right.$$ (4)

$$S_{j}=\sqrt{\frac{1}{m-1}\sum_{i=1}^{m}{\left({b^{\prime }}_{ij}-\bar{{b^{\prime }}_{j}}\right)}^{2}}$$ (5)

$$R_{j}=\sum_{j=1}^{n}(1-r_{ij})$$ (6)

$$G_{i}=S_{j}R_{j}$$ (7)

$$w_{c}=\frac{G_{i}}{\sum_{j=1}^{n}G_{j}}$$ (8)

In the equation, ${b^{\prime }}_{ij}$ is the normalized indicator value. The indicator variability is *S*_*j*_, where $\bar{{b^{\prime }}_{j}}=\frac{1}{m}\sum_{i=1}^{m}{b^{\prime }}_{ij}$. The metric conflict is *R*_*j*_. The amount of information is *G*_*i*_. *w*_*c*_ is the objective weight of each indicator obtained using the CRITIC method.

#### 4.2.2. The BWM method determines the subjective weight of indicators

The BWM method is a subjective empowerment method that can obtain consistent results with less comparative information. First determine the set of indicators, c = {*c*_1_,*c*_2_,⋯,*c*_*n*_} and determine the best indicator *c*_*b*_ and the worst indicator *c*_*d*_. Next, the ninescale method is used to compare the optimal indicator with the rest of the indicators to determine the weight of the optimal indicator relative to the other indicators: *A*_*b*_ = (*a*_*b*1_,*a*_*b*2_,⋯,*a*_*bn*_), where *a*_*bj*_ ∈ [1,9] indicates the importance of the optimal indicator compared to the indicator, where *a*_*bj*_ ∈ [1,9], *j* = 1, 2, ⋯*n*.

The weight of the other indicators relative to the worst indicators is determined using the ninescale method: *A*_*d*_ = (*a*_1*d*_, *a*_2*d*_,⋯, *a*_*nd*_), where *a*_*jd*_ indicates the importance of indicator *j* compared to the worst indicator, where *a*_*jd*_ ∈ [1,9], *j* = 1, 2,⋯*n*.

Finally, a linear model is constructed to find the minimum solution of $\left.\frac{w_{b}}{w_{j}}\right|a_{bj}$ and $\left.\frac{w_{j}}{w_{d}}\right|a_{jd}$ for the maximum values of all *j* ∈ {1, 2,⋯*n*}, and the subjective weight (*w*_1_, *w*_2_,⋯, *w*_*n*_) of each evaluation indicators is obtained by using Eq 9.

$$\begin{array}{l} \text{min}\;\xi \\ s.t\left\{\begin{array}{c} \left|\frac{w_{b}}{w_{j}}-a_{bj}\right|\leq \xi ,j=1,2,\cdots n\\ \left|\frac{w_{j}}{w_{d}}-a_{jd}\right|\leq \xi ,j=1,2,\cdots n\\ \sum_{j-1}^{n}w_{j}=1\\ w_{j}\geq 0,j=1,2,\cdots ,n \end{array}\right. \end{array}$$ (9)

where *a*_*bj*_ is the element in *A*_*b*_ and *a*_*jd*_ is the element in *A*_*d*_.

#### 4.2.3. Game theory combination empowerment

Based on the CRITIC method and BWM method, this paper uses game theory combination weighting to determine the weights of resilience indicators, aiming to ensure the coordination of the weights of each indicators of supply chain resilience and reduce the differences of each indicators. Firstly, the basic weight vector set W of supply chain resilience evaluation indicators is established. where n is the number of supply chain resilience evaluation indicators, t means that the indicators weight is calculated using the tth method, and S represents that the indicators weight is calculated using the S method. And this S vector is arbitrarily and linearly combined as:

$$w=\sum_{t=1}^{S}\lambda_{t}w_{t}^{T},\lambda_{t}>0$$ (10)

The principle of game theory is used to balance different weights, and the linear combination coefficient is optimized to minimize dispersion to obtain comprehensive weights, namely:

$$\text{min}\left\|\sum_{t=1}^{S}\lambda_{t}w_{t}^{T}-w_{t}\right\|,t=1,2,\cdots ,S$$ (11)

Transform Eq (12) equivalently into a system of linear equation that optimize first-derivative conditions:

$$\left[\begin{array}{cccc} w_{1}w_{1}^{T} & w_{1}w_{2}^{T} & \cdots & w_{1}w_{n}^{T}\\ w_{2}w_{1}^{T} & w_{2}w_{2}^{T} & \cdots & w_{2}w_{n}^{T}\\ \vdots & \vdots & \vdots & \vdots \\ w_{n}w_{1}^{T} & w_{n}w_{2}^{T} & \cdots & w_{n}w_{n}^{T} \end{array}\right]\left[\begin{array}{c} \lambda_{1}\\ \lambda_{2}\\ \vdots \\ \lambda_{n} \end{array}\right]=\left[\begin{array}{c} w_{1}w_{1}^{T}\\ w_{2}w_{2}^{T}\\ \vdots \\ w_{n}w_{n}^{T} \end{array}\right]$$ (12)

After solving, the optimal combination coefficient can be obtained λ* = (*λ*_1_, *λ*_2_, ⋯, *λ*_*n*_) Normalize the optimal weight coefficient:

$$\lambda_{t}^{*}=\frac{\left|\lambda_{t}\right|}{\sum_{t=1}^{S}\left|\lambda_{t}\right|}$$ (13)

The final weights are:

$$w=\sum_{t=1}^{S}\lambda_{t}^{*}w_{t}^{T},t=1,2,\cdots ,S$$ (14)

### 4.3. Improved extension integrated evaluation methods

Extenics is an original transversal discipline proposed by Chinese scholars in 1983, which combines the theory of matter elements with the theory of extension sets, often in a way that combines qualitative and quantitative methods to study and solve complex contradictory problems [57]. The use of matter element analysis theory to establish a comprehensive evaluation model of supply chain resilience can solve the problem of uncertainty and incompatibility of supply chain resilience evaluation factors. But the general extension comprehensive evaluation method also tends to have the problems of too large measured value of indicators, vague evaluation results and poor dynamic prediction. Aiming at the above problems, this paper introduces the nearness degree function to improve the matter element extension model, and proposes a new supply chain resilience evaluation model. The model can not only adjust the supply chain resilience evaluation system according to the actual needs of enterprises, but also overcome the problem that the measured values of indicators are beyond the range of the stanza domain and the correlation degree cannot be calculated. In addition, the dynamic prediction of elasticity indicators can be achieved through the eigenvalues of grade variables. Specifically, the improved matter element extension evaluation method mainly includes the following four steps.

Step 1: Determine the matter element
  The supply chain of low carbon enterprises is a "thing", its resilience index is a "feature", and the value of the supply chain resilience index is a "quantitative value". Therefore, supply chain resilience matter elements can be composed of three elements: "things", "characteristics" and "magnitude", which are denoted as R, R is ndimensional matter, and N, c, and v are called the three elements of matter. Assuming that the object to be evaluated is *N*_*y*_, the supply chain resilience evaluation object is expressed as:
$$R=\left(N,c,v\right)=\left[\begin{array}{ccc} N & c_{1} & v_{1}\\ & c_{2} & v_{2}\\ & \vdots & \vdots \\ & c_{n} & v_{n} \end{array}\right]$$ (15)
  Among them, the evaluation indicators *c* = (*c*_1_, *c*_2_, ⋯, *c*_*n*_) and the indicator measure value *v* = (*v*_1_, *v*_2_, ⋯, *v*_*n*_).
Step 2: Determine the classic and stanza domains
  The classic domain is determined according to the range of the magnitude value corresponding to each evaluation indicators under different levels of resilience of supply chain resilience. Assuming that supply chain resilience can be divided into m levels, the established classical domain matter element is shown in Eq (16).
$$R_{j}=\left(N_{j},c_{i},v_{ij}\right)=\left[\begin{array}{ccc} N_{j} & c_{1} & v_{1j}\\ & c_{2} & v_{2j}\\ & \vdots & \vdots \\ & c_{n} & v_{nj} \end{array}\right]=\left[\begin{array}{ccc} N_{j} & c_{1} & \langle a_{1j},b_{1j}\rangle \\ & c_{2} & \langle a_{2j},b_{2j}\rangle \\ & \vdots & \vdots \\ & c_{n} & \langle a_{nj},b_{nj}\rangle \end{array}\right]$$ (16)
  where *R*_*j*_ represents the classical domain matter, and *N*_*j*_ represents the jth level of supply chain resilience, *j* = 1, 2, ⋯, *m*. *c*_*i*_ represents the ith evaluation indicator, *i* = 1, 2, ⋯, *n*. *v*_*ij*_ represents the value range of *c*_*i*_ corresponding to the ith evaluation indicators when the supply chain resilience level is j, where *v*_*ij*_ can be represented by (*a*_*ij*_, *b*_*ij*_).
  Its stanza domain *R*_*p*_ is constructed through the classic domain, and *R*_*p*_ ⊃ *R*_*j*_.
$$R_{p}=\left(N_{p},c_{i},v_{ip}\right)=\left[\begin{array}{ccc} N_{p} & c_{1} & v_{1p}\\ & c_{2} & v_{2p}\\ & \vdots & \vdots \\ & c_{n} & v_{np} \end{array}\right]=\left[\begin{array}{ccc} N_{p} & c_{1} & \langle a_{p1},b_{p1}\rangle \\ & c_{2} & \langle a_{p2},b_{p2}\rangle \\ & \vdots & \vdots \\ & c_{n} & \langle a_{pn},b_{pn}\rangle \end{array}\right]$$ (17)
  where *N*_*p*_ represents the level of resilience for all supply chains. *v*_*ip*_ represents the value range of *c*_*i*_ under the resilience level of the entire supply chain, *v*_*ip*_ is the union of all *v*_*ij*_, and *v*_*ip*_ = 〈*a*_*pi*_, *b*_*pi*_〉, *a*_*pi*_, and *b*_*pi*_ can be used to be the upper and lower limits of the stanza domain, where *i* = 1, 2, ⋯, *n*. *j* = 1, 2, ⋯, *m*.
Step 3: Standardization
  When the value of the evaluation indicators exceeds the stanza domain boundary, the evaluation indicators needs to be standardized. The maximum value normalization is processed as follows:
$${v^{\prime }}_{i}=\frac{v_{i}-a_{pi}}{b_{pi}-a_{pi}}$$ (18)
  The minimum specification is processed as follows:
$${v^{\prime }}_{i}=\frac{b_{pi}-v_{i}}{b_{pi}-a_{pi}}$$ (19)
  The improved maxima classical domain object matrix is:
$${R^{\prime }}_{j}=\left[\begin{array}{ccc} N_{j} & c_{1} & \langle \frac{a_{1j}-a_{p1}}{b_{p1}-a_{p1}},\frac{b_{1j}-a_{p1}}{b_{p1}-a_{p1}}\rangle \\ & \vdots & \vdots \\ & c_{n} & \langle \frac{a_{nj}-a_{pn}}{b_{pn}-a_{pn}},\frac{b_{nj}-a_{pn}}{b_{pn}-a_{pn}}\rangle \end{array}\right]$$ (20)
  The improved minima classical domain object matrix is:

$${R^{\prime }}_{j}=\left[N_{j},c_{i},{v^{\prime }}_{ij}\right]=\left[\begin{array}{ccc} N_{j} & c_{1} & \langle \frac{b_{p1}-b_{1j}}{b_{p1}-a_{p1}},\frac{b_{p1}-a_{1j}}{b_{p1}-a_{p1}}\rangle \\ & \vdots & \vdots \\ & c_{n} & \langle \frac{b_{pn}-b_{nj}}{b_{pn}-a_{pn}},\frac{b_{pn}-a_{nj}}{b_{pn}-a_{pn}}\rangle \end{array}\right]$$ (21)

Step 4: Determine the nearness degree
  Because when evaluating the level of matter, the extendable model may have the problem of blurring its own boundaries, resulting in distortion of evaluation results. Therefore, this article uses nearness degree to optimize it. Nearness degree is a concept in fuzzy mathematics that indicates the degree of similarity between two fuzzy subsets. The nearness degree calculation equation selected for this study is as follows:
$$N\left(t_{iy}\right)=1-\frac{1}{n(n+1)}\sum_{i=1}^{n}D_{j}({v^{\prime }}_{yi})w_{i}*$$ (22)
  where *N*(*t*_*iy*_) is the object to be evaluated y on the nearness degree of each supply chain resilience indicators on the supply chain resilience level j. n is the number of indicators, $w_{i}^{*}$ represents the comprehensive weight value of the ith evaluation indicators, and $D_{j}(v_{yi}^{\prime })$ represents the distance between the standardized ith evaluation indicators and the jth evaluation level classic domain $\langle {a^{\prime }}_{ij},{b^{\prime }}_{ij}\rangle$.
$$D_{j}({v^{\prime }}_{yi})=\left|v_{yi}-\frac{{a^{\prime }}_{ij}+{b^{\prime }}_{ij}}{2}\right|-\frac{{b^{\prime }}_{ij}-{a^{\prime }}_{ij}}{2}$$ (23)
  Next, the nearness degree is normalized to increase the distinction between the matter to be evaluated and the nearness degree of each evaluation level, so that the evaluation level can be determined more accurately.
$$N^{\prime }\left(t_{iy}\right)=\frac{N\left(t_{iy}\right)-\text{min}\{N\left(t_{iy}\right)\}}{\text{max}\{N\left(t_{iy}\right)\}-\text{min}\{N\left(t_{iy}\right)\}}$$ (24)
  The affiliation level of the object y to be evaluated is written as:
$$L\left(N_{y}\right)=\text{max}\left\{N^{\prime }\left(N_{y}\right)\right\}\left(j=1,2,\cdots ,m\right)$$ (25)
$$j^{*}=\frac{\sum_{j=1}^{m}jN^{\prime }\left(t_{iy}\right)}{\sum_{j=1}^{m}N^{\prime }\left(t_{iy}\right)}$$ (26)
  j* is the characteristic value of the grade variable of the object y to be evaluated, and the degree to which the thing to be evaluated is biased towards the adjacent risk level can be judged by j*.

## 5. Examples analysis

### 5.1. Determine the weights of supply chain resilience evaluation indicators

Enterprise X is a low carbon manufacturing enterprise integrating research and development, production, sales and service, and is an important enterprise in the field of machinery. In order to accurately assess the level of supply chain resilience of Enterprise X, five experts are selected to evaluate each index of supply chain resilience of X low-carbon enterprise, which are represented by Expert A, Expert B, Expert C, Expert D, and Expert E respectively. Due to the complexity of the project and the ambiguity of the information, the results of the fuzzy envelope calculations corresponding to the use of single and comparative languages by the expert panel are summarised in Table 2. S1 Table collects the results of the expert panel’s assessment. After evaluation by the expert panel, the results can be summarised in the matrix D.

**Table 2:** Fuzzy envelope of composite languages.

Language set	Composite language	Blurred envelopes	The number of gradient blurs
Single language	weak	(0,0,0,0.5)	0.06
	Weaker	(0.0.25,0.25,0.5)	0.25
	average	(0.25,0.5,0.5,0.75)	0.5
	Stronger	(0.5,0.75,0.75,1)	0.75
	Strong	(0.75,1,1,1)	0.94
Compare languages	At least weaker	(0,0.33,1,1)	0.58
	At least average	(0.25,0.69,1,1)	0.74
	At least stronger	(0.5,0.94,1,1)	0.86
	At most Weaker	(0,0,0.063,0.5)	0.14
	At most average	(0,0,0.31,0.75)	0.27
	At most stronger	(0,0,0.67,1)	0.42
	Between weak and weaker	(0.0,0.25,0.5)	0.19
	Between weak and average	(0.0.17,0.33,0.75)	0.31
	Between weaker and average	(0.0.25,0.5,0.75)	0.38
	Between weaker and stronger	(0.0.42,0.58,1)	0.5
	Between average and stronger	(0.25,0.5,0.75,1)	0.63
	Between average and strong	(0.25,0.58,0.92,1)	0.69
	Between stronger and strong	(0.5,0.75,1,1)	3.25


$$D_{25\times 5}=\left[\begin{array}{ccccc} \left(0.25,0.58,0.92,1\right) & \left(0.5,0.75,0.75,1\right) & \left(0.25,0.69,1,1\right) & \left(0,0,0.67,1\right) & \left(0.5,0.94,1,1\right)\\ \left(0.25,0.5,0.5,0.75\right) & \left(0.25,0.25,0.5,0.75\right) & \left(0,0,0.67,1\right) & \left(0,0.42,0.58,1\right) & \left(0,0.33,1,1\right)\\ \vdots & \vdots & \vdots & \vdots & \vdots \\ \left(0,0.25,0.25,0.5\right) & \left(0.25,0.5,0.5,0.75\right) & \left(0,0,0.06,0.5\right) & \left(0.25,0.5,0.5,0.75\right) & \left(0,0,0.31,0.75\right) \end{array}\right]$$ (27)

Relevant weights are given to the authority of different experts in the field. Empower A, B, C, D, and E experts as *w*_*t*_ = (0.25, 0.1, 0.3, 0.2, 0.15). A weighted fuzzy matrix D′ can be obtained.


$${D^{\prime }}_{25\times 5}=\left[\begin{array}{ccccc} \left(0.06,0.15,0.23,0.25\right) & \left(0.05,0.08,0.08,0.1\right) & \left(0.08,0.2,0.3,0.3\right) & \left(0,0,0.13,0.2\right) & \left(0.08,0.14,0.15,0.15\right)\\ \left(0.06,0.13,0.13,0.19\right) & \left(0.03,0.03,0.05,.08\right) & \left(0,0,0.2,0.3\right) & \left(0,0.08,0.12,0.2\right) & \left(0,0.05,0.15,0.15\right)\\ \vdots & \vdots & \vdots & \vdots & \vdots \\ \left(0,0.06,0.06,0.13\right) & \left(0.03,0.05,0.05,0.08\right) & \left(0,0,0.02,0.15\right) & \left(0.05,0.1,0.1,0.15\right) & \left(0,0,0.05,0.11\right) \end{array}\right]$$ (28)

Next, the weighted fuzzy matrix *D*′ is processed by Eq (1), and the evaluation matrix R can be obtained.


$$R_{5\times 25}=\left[\begin{array}{cccccccc} 0.1725 & 0.125 & 0.185 & 0.235 & 0.105 & 0.105 & \cdots & 0.0625\\ 0.075 & 0.038 & 0.075 & 0.069 & 0.074 & 0.05 & \cdots & 0.05\\ 0.222 & 0.126 & 0.975 & 0.189 & 0.222 & 0.15 & \cdots & 0.042\\ 0.084 & 0.1 & 0.188 & 0.138 & 0.1 & 0.126 & \cdots & 0.1\\ 0.129 & 0.021 & 0.111 & 0.075 & 0.1035 & 0.111 & \cdots & 0.0405 \end{array}\right]$$ (29)

After obtaining the evaluation matrix, the CRITIC weights of each indicators of supply chain resilience were obtained by using Eqs (2)–(9). And through the formula (10) and (11), the BWM method is used to determine the subjective weight of the toughness indicators. Finally, through the method of game theory combined weighting, the results of subjective weights and objective weights are brought into the formula (12)–(16) to determine the preference coefficients of subjective and objective weights, which are (0.06, 0.94), respectively, so as to obtain the comprehensive weights of each indicators as shown in Table 3.

**Table 3:** Weight of supply chain resilience indicators.

Indicators	CRITIC weight	BWM weight	Game weights	Indicators	CRITIC weight	BWM weight	Game weights	Indicators	CRITIC weight	BWM weight	Game weights
c_1_	1.66	2.711	1.723	c_19_	2.066	0.377	1.965	c_37_	1.487	0.300	1.416
c_2_	1.653	2.711	1.716	c_20_	1.69	1.318	1.668	c_38_	1.623	0.901	1.580
c_3_	2.087	5.422	2.287	c_21_	1.697	4.283	1.852	c_39_	1.862	0.501	1.780
c_4_	1.849	5.422	2.063	c_22_	1.989	1.318	1.949	c_40_	2.001	1.546	1.974
c_5_	1.636	10.843	2.188	c_23_	1.778	2.306	1.810	c_41_	1.656	0.476	1.585
c_6_	2.14	1.581	2.106	c_24_	1.625	1.581	1.622	c_42_	1.921	0.832	1.856
c_7_	1.687	2.937	1.762	c_25_	1.946	0.678	1.870	c_43_	1.873	4.280	2.017
c_8_	1.987	0.904	1.922	c_26_	1.808	2.306	1.838	c_44_	1.924	4.280	2.065
c_9_	2.67	1.632	2.608	c_27_	1.847	4.283	1.993	c_45_	1.702	2.854	1.771
c_10_	1.718	0.502	1.645	c_28_	2.032	1.318	1.989	c_46_	1.997	1.902	1.991
c_11_	1.892	0.879	1.831	c_29_	1.885	2.636	1.930	c_47_	1.582	0.527	1.519
c_12_	2.297	0.661	2.199	c_30_	1.777	2.636	1.829	c_48_	1.506	0.922	1.471
c_13_	2.097	3.379	2.174	c_31_	1.642	0.904	1.598	c_49_	2.002	1.713	1.985
c_14_	2.566	1.175	2.483	c_32_	1.702	0.602	1.636	c_50_	1.713	0.633	1.648
c_15_	2.606	0.808	2.498	c_33_	2.688	3.397	2.731	c_51_	1.482	0.271	1.409
c_16_	2.066	3.614	2.159	c_34_	1.649	1.288	1.627	c_52_	1.817	1.581	1.803
c_17_	1.706	2.410	1.748	c_35_	2.38	0.586	2.272	c_53_	1.789	1.581	1.777
c_18_	1.502	1.318	1.491	c_36_	2.042	0.200	1.931				

### 5.2. Steps for evaluating the resilience of the supply chain

#### 5.2.1. Determination of supply chain resilience grade, classical domain and stanza domain

Through reviewing relevant literature and consulting experts’ opinions on the grade division of supply chain resilience level of low carbon enterprises, the supply chain resilience level is divided into four levels, which are excellent, good, general and poor from high to low, corresponding to the following: I (poor), II (general), III (good), IV (excellent). Based on the above identification of supply chain resilience grade division, this paper’s supply chain resilience quantitative evaluation indexes at each level is shown in Table 4, qualitative indexes of each classical domain grade corresponds to I (0~60), II (60~80), III (80~90), IV (90~100).

**Table 4:** Classical domains for quantitative evaluation indicator.

Indicators	Description of indicators	The classic domain takes the value interval			
		I (poor)	II (medium)	III (good)	IV (excellent)
c_1_	Product range and quantity	[5,11)	[11,15)	[15,20)	[20,30]
c_3_	Response time to customer needs/industry average response time	[0.65,0.98]	[0.51,0.65)	[0.48,0.51)	[0.37,0.48)
c_4_	Reduced delivery time/total total delivery time	[0.09,0.29)	[0.29,0.59)	[0.59,0.76)	[0.76,0.85]
c_6_	Number of order delivery failures/total number of orders	[0.222,0.333]	[0.166,0.222)	[0.056,0.166)	[0,0.056)
c_7_	Number of order delivery delays/total number of orders	[0.315,0.421]	[0.215,0.315)	[0.156,0.215)	[0.053,0.156)
c_8_	Number of orders provided in advance/total number of orders	[0.55,0.65)	[0.65,0.75)	[0.75,0.85)	[0.85,0.94]
c_16_	Troublefree operation time of production equipment/Total operation time	[0.32,0.51)	[0.51,0.63)	[0.63,0.71)	[0.71,0.98]
c_17_	Remaining capacity of production equipment/maximum capacity	[0.3,0.4]	[0.2,0.3)	[0.1,0.2)	[0,0.1)
c_28_	Number of collaborative partners that can enter quickly + number of collaborative partners that can exit quickly	[5,9)	[9,14)	[14,17)	[17,20]
c_31_	Information acquisition time + processing time + feedback time	[9,14]	[7,9)	[5,7)	[3,5)
c_32_	Total number of messages to which the enterprise can respond effectively / number of members to which the enterprise can connect	[0.3,0.5)	[0.5,0.7)	[0.7,0.8)	[0.8,0.9]
c_33_	Number of tracked shipping messages/total number of orders	[0.667,0.778)	[0.778,0.834)	[0.834,0.944)	[0.944,1]
c_34_	Number of accurate messages / Number of all messages	[0.42,0.6)	[0.6,0.77)	[0.77,0.84)	[0.84,0.92]
c_36_	Cost of using funds / (total financing financing costs)	[0.5.0.7]	[0.4,0.5)	[0.3,0.4)	[0.1,0.3)
c_40_	Gross profit / main business revenue	[0.01,0.04)	[0.04,0.15)	[0.15,0.17)	[0.17,0.25]
c_41_	Growth in sales revenue / sales revenue	[0.01,0.04)	[0.04,0.15)	[0.15,0.17)	[0.17,0.25]
c_42_	360 / number of inventory turns	[100,120]	[80,100)	[60,80)	[30,60)
c_43_	Average market price actual cost of product	[87,119)	[119,200)	[200,279)	[279,360]
c_44_	Average market price of products product selling price	[0,10)	[10,20)	[20,30)	[30,40]
c_45_	Volume of products sold/volume of similar products sold in the market	[0.01,0.04)	[0.04,0.08)	[0.08,0.12)	[0.12,0.14]
c_49_	Amount of knowledge shared in a timely and accurate manner / Amount of knowledge that should be shared	[0.23,0.49)	[0.49,0.64)	[0.64,0.82)	[0.82,0.94]
c_52_	Cost of developing a new product	[17,24]	[13,17)	[9,13)	[7,9)
c_53_	Time required to develop a new product	[4,5]	[3,4)	[2,3)	[1,2)

#### 5.2.2. Raw data and standardization of the indicator to be evaluated

The supply chain resilience evaluation system constructed in this paper has a total of 53 secondary indicators, including 29 qualitative indicators and 24 quantitative indicators. When assessing the supply chain resilience of a low carbon enterprise, quantifiable indicators are quantitatively counted, and five experts A, B, C, D and E can be invited to score the supply chain resilience evaluation indicators and quantify the qualitative indicators. After collecting the relevant data of low carbon enterprises, the raw data of each indicator can be obtained. The original data of the low carbon enterprise was standardized using Formula (18)–(21), and the results are shown in Table 5.

**Table 5:** Raw data and standardization of the indicators to be evaluated.

Indicators	raw data	Specification of indicators	Classic domain specification			
			I(poor)	II(average)	III(good)	IV(excellent)
c_1_	13	0.32	[0,0.24]	[0.24,0.4]	[0.4,0.6]	[0.6,1]
c_2_	77.8	0.78	[0,0.6)	[0.6,0.8)	[0.8,0.9)	[0.9,1]
c_3_	0.49	0.8	[0,0.54]	[0.54,0.77]	[0.77,0.82]	[0.82,1]
c_4_	0.78	0.91	[0,0.23]	[0.23, 0.66]	[0.66,0.88]	[0.88,1]
c_5_	75.45	0.75	[0,0.6)	[0.6,0.8)	[0.8,0.9)	[0.9,1]
c_6_	0.184	0.48	[0,0.33]	[0.33,0.5]	[0.5,0.83]	[0.83,1]
c_7_	0.245	0.48	[0,0.28]	[0.29,0.56]	[0.56,0.72]	[0.72,1]
c_8_	0.675	0.56	[0,0.26]	[0.26,0.51]	[0.51,0.77]	[0.77,1]
c_9_	88.2	0.88	[0,0.6)	[0.6,0.8)	[0.8,0.9)	[0.9,1]
c_10_	64.3	0.64	[0,0.6)	[0.6,0.8)	[0.8,0.9)	[0.9,1]
c_11_	76.45	0.76	[0,0.6)	[0.6,0.8)	[0.8,0.9)	[0.9,1]
c_12_	77.9	0.78	[0,0.6)	[0.6,0.8)	[0.8,0.9)	[0.9,1]
c_13_	75.6	0.76	[0,0.6)	[0.6,0.8)	[0.8,0.9)	[0.9,1]
c_14_	75.4	0.75	[0,0.6)	[0.6,0.8)	[0.8,0.9)	[0.9,1]
c_15_	78.5	0.79	[0,0.6)	[0.6,0.8)	[0.8,0.9)	[0.9,1]
c_16_	0.68	0.55	[0,0.29]	[0.29,0.47]	[0.47,0.59]	[0.59,1]
c_17_	0.26	0.35	[0,0.25]	[0.25,0.5]	[0.5,0.75]	[0.75,1]
c_18_	80.55	0.81	[0,0.6)	[0.6,0.8)	[0.8,0.9)	[0.9,1]
c_19_	76.95	0.77	[0,0.6)	[0.6,0.8)	[0.8,0.9)	[0.9,1]
c_20_	79	0.79	[0,0.6)	[0.6,0.8)	[0.8,0.9)	[0.9,1]
c_21_	74.6	0.75	[0,0.6)	[0.6,0.8)	[0.8,0.9)	[0.9,1]
c_22_	71.8	0.72	[0,0.6)	[0.6,0.8)	[0.8,0.9)	[0.9,1]
c_23_	81.7	0.82	[0,0.6)	[0.6,0.8)	[0.8,0.9)	[0.9,1]
c_24_	86.95	0.87	[0,0.6)	[0.6,0.8)	[0.8,0.9)	[0.9,1]
c_25_	82.95	0.83	[0,0.6)	[0.6,0.8)	[0.8,0.9)	[0.9,1]
c_26_	83.2	0.83	[0,0.6)	[0.6,0.8)	[0.8,0.9)	[0.9,1]
c_27_	74.4	0.74	[0,0.6)	[0.6,0.8)	[0.8,0.9)	[0.9,1]
c_28_	25	1.33	[0,0.267)	[0.267,0.6)	[0.6,0.8)	[0.8,1]
c_29_	77.6	0.78	[0,0.6)	[0.6,0.8)	[0.8,0.9)	[0.9,1]
c_30_	85.7	0.86	[0,0.6)	[0.6,0.8)	[0.8,0.9)	[0.9,1]
c_31_	6	0.73	[0,0.45)	[0.45,0.64)	[0.64,0.82)	[0.82,1]
c_32_	0.6	0.5	[0,0.33)	[0.33,0.67)	[0.67,0.83)	[0.83,1]
c_33_	0.796	0.39	[0,0.33)	[0.33,0.5)	[0.5,0.83)	[0.83,1]
c_34_	0.79	0.74	[0,0.36)	[0.36,0.7)	[0.7,0.84)	[0.84,1]
c_35_	84.7	0.85	[0,0.6)	[0.6,0.8)	[0.8,0.9)	[0.9,1]
c_36_	0.13	0.95	[0,0.33]	[0.33,0.5]	[0.5,0.67]	[0.67,1]
c_37_	87	0.87	[0,0.6)	[0.6,0.8)	[0.8,0.9)	[0.9,1]
c_38_	82.1	0.82	[0,0.6)	[0.6,0.8)	[0.8,0.9)	[0.9,1]
c_39_	76.4	0.76	[0,0.6)	[0.6,0.8)	[0.8,0.9)	[0.9,1]
c_40_	0.16	0.62	[0,0.13]	[0.13,0.58)	[0.58,0.67)	[0.67,1]
c_41_	0.09	0.33	[0,0.13]	[0.13,0.58)	[0.58,0.67)	[0.67,1]
c_42_	92	0.31	[0,0.22]	[0.22,0.44)	[0.44,0.67)	[0.67,1]
c_43_	190	0.38	[0,0.12]	[0.12,0.41)	[0.41,0.7)	[0.7,1]
c_44_	22	0.55	[0,0.25]	[0.25,0.5]	[0.5,0.75]	[0.75,1]
c_45_	0.07	0.46	[0,0.23]	[0.23,0.54]	[0.54,0.85]	[0.85,1]
c_46_	76.5	0.77	[0,0.6)	[0.6,0.8)	[0.8,0.9)	[0.9,1]
c_47_	74.3	0.74	[0,0.6)	[0.6,0.8)	[0.8,0.9)	[0.9,1]
c_48_	78.6	0.79	[0,0.6)	[0.6,0.8)	[0.8,0.9)	[0.9,1]
c_49_	0.74	0.72	[0,0.37)	[0.37,0.58)	[0.58,0.83)	[0.83,1]
c_50_	82.1	0.82	[0,0.6)	[0.6,0.8)	[0.8,0.9)	[0.9,1]
c_51_	83.4	0.83	[0,0.6)	[0.6,0.8)	[0.8,0.9)	[0.9,1]
c_52_	19	0.29	[0,0.41)	[0.41,0.65)	[0.65,0.88)	[0.88,1]
c_53_	5	0	[0,0.25]	[0.25,0.5]	[0.5,0.75]	[0.75,1]

#### 5.2.3. Multilevel extendable evaluation

The weight of each resilience evaluation indicates can be obtained from Table 1, and after obtaining the data of low carbon enterprise supply chain resilience index specification processing, they are brought into the Eqs (22) to (26), and the nearness degree of the evaluation indexes at all levels of the resilience of the supply chain of this low carbon enterprise can be obtained, as shown in Tables 6–8. Through the size of the nearness degree can judge the resilience grade of each indicator, and through the grade variable eigenvalue can judge the degree of the things to be evaluated to be biased towards the adjacent grades.

**Table 6:** Proximity and grade of secondary indicators of supply chain resilience evaluation.

Indicators	The grading progress of each secondary resilience indicators				grade	Rank variable feature values	Trends in risk levels
	I(poor)	II(average)	III(good)	IV(excellent)			
B_1_	0.978	1.008	0.992	0.967	average	2.17	Convert to good
B_2_	0.923	0.978	0.999	1.00	excellent	3.11	Convert to good
B_3_	0.925	1.025	0.975	0.925	average	2.33	Convert to good
B_4_	0.975	1.001	0.997	0.977	average	2.52	Convert to good
B_5_	0.985	0.999	0.999	0.990	average	2.72	Convert to good
B_6_	0.996	1.001	0.999	0.994	average	2.19	Convert to good
B_7_	0.965	1.004	0.996	0.957	average	2.34	Convert to good
B_8_	0.984	1.001	0.999	0.993	average	2.79	Convert to good
B_9_	0.986	1.003	0.997	0.990	average	2.58	Convert to good
B_10_	0.959	0.992	1.005	0.995	good	3.03	Convert to excellent
B_11_	0.960	0.980	0.984	0.985	excellent	3.07	Convert to good
B_12_	0.964	0.997	1.002	0.986	good	2.89	Convert to average
B_13_	0.963	1.007	0.993	0.965	average	2.45	Convert to good
B_14_	0.983	1.000	1.000	0.983	good	2.49	Convert to average
B_15_	0.984	0.993	0.996	0.998	excellent	3.14	Convert to good
B_16_	0.978	1.006	0.994	0.980	average	2.45	Convert to good
B_17_	0.953	0.998	1.002	0.969	good	2.74	Convert to average
B_18_	0.967	1.009	0.991	0.958	average	2.26	Convert to good
B_19_	0.980	0.997	1.002	0.990	good	2.84	Convert to average
B_20_	0.963	0.996	1.004	0.996	good	3.00	good
B_21_	1.010	0.969	0.928	0.888	poor	1.66	Convert to average

**Table 7:** Supply chain resilience evaluation level 1 indicators proximity and grade.

Indicators	grade				grade	Rank variable feature values	Grade change trend
	I (poor)	II (average)	III (good)	IV (excellent)			
A_1_	0.9960	0.9998	0.9995	0.9977	average	2.77	Convert to good
A_2_	0.9990	1.0001	0.9999	0.9991	average	2.50	Convert to good
A_3_	0.9957	0.9987	0.9989	0.9983	good	2.95	Convert to average
A_4_	0.9962	1.0001	0.9999	0.9969	average	2.62	Convert to good
A_5_	0.9977	0.9998	0.9995	0.9987	average	2.76	Convert to good
A_6_	0.9975	0.9986	0.9979	0.9952	average	2.06	Convert to good

**Table 8:** Supply chain resilience evaluation indicators proximity and grade.

Indicators	I (poor)	II (average)	III (good)	IV (excellent)	grade	Rank variable feature values	Grade change trend
*A*	0.9999	1.0000	1.0000	0.9999	average	2.69	Convert to good

### 5.3. Analysis of results

The overall evaluation results of the level of supply chain resilience in Table 8 shows that the supply chain resilience level of the evaluated low-carbon enterprises is average. But due to the eigenvalue of the grade variable j* = 2.69 > 2. This indicates that the supply chain resilience of this low carbon enterprise is moving towards a good grade. This shows that the current strategy of Enterprise X helps to improve the supply chain resilience.

The data in Table 7 is carried out to obtain the first level indicators affecting the supply chain resilience. After analysing the product supply resilience A1, resource resilience A2, information response resilience A4, financial resilience A5, and knowledge resilience A6, it can be found that although A1, A2, A4, A5 and A6 are out of the medium grade, but due to the relationship of the eigenvalue of their grade variable j* = 2.77 > 2.76 > 2.62 > 2.5 > 2.06 > 2, which indicates that the enterprise’s ability of absorbing, integrating, transforming, and applying the external knowledge in order to realize the innovation is poor, therefore, the Firms need to improve their knowledge resilience by enhancing their learning organisation, collaborative innovation capabilities and product development capabilities. This will not only promote communication among supply chain members, but also enhance the core competitiveness of enterprises and improve the resilience of the supply chain.

Analysing the data in Table 6 can lead to the secondary indicators that affect the level of supply chain resilience. By analysing the posting progress of each resilience level 2 evaluation indicator, it can be found that the product development B21 of the supply chain of the low carbon enterprise X has a grade of poor. This indicates that the low-carbon enterprise’s ability to develop products is weak, and it is also difficult for the enterprise to launch new products to meet consumer demand within a short period of time after perceiving market opportunities. However, the eigenvalue of the grade variable j* = 1.66 > 1, indicates that the company has now noticed this problem and is paying more attention to its product development capability to meet the market demand. In addition, among the resilience indicators with a general grade, all resilience indicators are shifting to a good grade. However, the slowest trend of transformation is the range of product supply and market position, which indicates that the future improvement aspects of this low carbon enterprise not only need to increase the variety and quantity of products to meet the personalised needs of consumers, but also to enhance the brand awareness of the product in the market as well as the market share.

## 6. Summary

### 6.1. Conclusions

The contribution of this paper is to propose a supply chain resilience evaluation method for low carbon enterprises based on fuzzy set theory and matter element extension theory, combined with the status quo of low carbon enterprises, with a combination of empowerment-improved matter element extension model. The method not only reduces the interference of internal and external environments on the opinions of decision makers, but also provides a dynamic early warning of supply chain resilience changes through the model. The main findings of this paper are as follows:

This paper divides the evaluation indexes of supply chain resilience based on quantitative and qualitative perspectives, and establishes a more perfect evaluation system of supply chain resilience indexes for low carbon enterprises. In the assignment of supply chain resilience indicators, the OWA operator was introduced to collect expert opinions, compared with the general expert scoring method, this method can fully express the expert’s opinion, and the use of a single language and comparative language can effectively reduce the amount of fuzzy envelope calculation. In addition, when using the game theory combination assignment method to assign indicator weights, not only can it fully take into account the complexity and correlation between the indicators in full consideration, but also reduces the interference of subjective preferences, and solves the problem of absolute objectivity or overly subjective when assigning indicators. The accuracy of the evaluation results is improved.

In terms of the evaluation method, the use of the asymmetric nearness degree principle instead of the maximum affiliation principle not only solves the problem of distortion of the evaluation results due to the ambiguity of the boundaries, but also solves the problem of evaluating the indicators exceeding the stanza domain, and improves the accuracy and applicability of the method. Compared with the general evaluation method, the improved matter element extension model can predict the change trend of supply chain resilience by using the variable eigenvalues, which provides assistance for low carbon enterprises to establish supply chain risk early warning models and helps to improve the efficiency of resource allocation in low carbon enterprises. In addition, this method can be extended to other industries and provide relevant references for similar evaluation problems.

The supply chain resilience evaluation model proposed in this study enables real time prediction of changes in resilience metrics, which provides assistance in the assessment and early warning of supply chain resilience in low carbon enterprises and reduces the risk of disruptions in uncertain environments for low carbon enterprises. However, the limitation of this study is that the model relies on the collection of resilience indicator data. Therefore, future research should focus on obtaining the required data through multiple channels. Meanwhile, there is a time lag in the process of strategy adjustment for low carbon firms. Therefore, future research needs to focus on the changes in resilience indicators over a short period of time so that firms can react more quickly to external risk shocks.

### 6.2. Revelation

This study explores a new supply chain resilience evaluation model based on hesitant fuzzy linguistic information and improved matter element topable model. The model makes up for the shortcomings of supply chain resilience evaluation methods in the field of fuzzy set research, realises dynamic early warning of supply chain resilience, and improves the theoretical system of supply chain resilience evaluation. In addition, this study analyses the impact of six key elements on supply chain resilience, and the results are conducive to helping low-carbon enterprises focus on the core elements of supply chain resilience, promoting mutual cooperation among enterprises in the supply chain, reducing supply chain vulnerability, and enhancing the resilience level and profitability of the supply chain. Meanwhile, the supply chain resilience evaluation model established in this study not only provides a reference for decision makers of relevant enterprises to establish a perfect resilience early warning system. And it provides help for the sustainable and healthy development of enterprises.

### 6.3. Research limitations and perspectives

This study mainly has the following limitations: (1) The model relies on the collection of resilience indicator data, which may affect the evaluation results of supply chain resilience if the data collection is incomplete. (2) There is a certain time lag in the process of strategy adjustment by low-carbon enterprises. Therefore, the coping strategies obtained based on the evaluation results of supply chain resilience can only play a more obvious role in the short term. (3) The conclusions of this study are somewhat homogeneous and lack comparative studies for other industries.

In the future, scholars can focus on obtaining the required data through multiple channels. In addition, in order to reduce the time lag of enterprises in adjusting their strategies, it is important to focus on the data changes of resilience indicators in a short period of time, so that enterprises can react more quickly when they are subject to external risk shocks. In addition, based on the supply chain resilience evaluation model in this study, supply chains in other industries can be studied in a targeted manner and relevant recommendations can be given.

## Supporting information

S1 Table Comparative language evaluation results among experts. (DOCX)
