# Supplementary material for: Resilience evaluation of low-carbon supply chain based on improved matter-element extension model
Source: PLoS One. 2024 Apr 1;19(4):e0301390. doi: 10.1371/journal.pone.0301390 (PMC10984551; doi:10.1371/journal.pone.0301390)
Supplement: S1 Table — (DOCX) [file pone.0301390.s001.docx]

Comparative language evaluation results among experts

| Indicators | $A$ | $B$ | $C$ | $D$ | $E$ |
| --- | --- | --- | --- | --- | --- |
| c_1_ | Between average and strong | stronger | At least average | At most stronger | At least stronger |
| c_2_ | average | Between weaker and average | At most stronger | Between weaker and stronger | At least weaker |
| c_3_ | At most average | average | Between weaker and average | average | At most average |
| c_4_ | strong | Between average and strong | Between average and stronger | Between average and strong | average |
| c_5_ | Between weak and weaker | At least weaker | At most average | average | weak |
| c_6_ | At most average | weak | Between weak and weaker | At most Weaker | weaker |
| c_7_ | weaker | average | At most Weaker | average | At most average |
| c_8_ | Between average and stronger | strong | stronger | At most stronger | Between weaker and stronger |
| c_9_ | stronger | At least average | Between average and strong | At least average | Between average and strong |
| c_10_ | average | weaker | Between weaker and average | Between weaker and average | average |
| c_11_ | average | Between average and stronger | Between average and strong | Between weaker and stronger | At least average |
| c_12_ | stronger | At most stronger | Between average and stronger | At least average | At most stronger |
| c_13_ | Between average and stronger | Between average and strong | average | At least average | At least average |
| c_14_ | At least average | stronger | average | average | Between average and strong |
| c_15_ | At least average | stronger | average | At least average | At most stronger |
| c_16_ | At most stronger | At least average | At least average | average | Between average and strong |
| c_17_ | At most stronger | average | Between weaker and stronger | Between average and stronger | At least average |
| c_18_ | At most stronger | Between average and stronger | strong | Between average and strong | Between average and stronger |
| c_19_ | At least average | At most stronger | Between weaker and average | Between average and stronger | At most average |
| c_20_ | average | At most average | strong | Between weak and weaker | At most average |
| c_21_ | At most average | average | stronger | Between average and stronger | At least average |
| c_22_ | average | Between average and stronger | At most stronger | Between average and strong | stronger |
| c_23_ | weaker | At most average | Between weaker and stronger | Between average and strong | average |
| c_24_ | Between average and strong | At most stronger | Between average and strong | At least average | Between average and stronger |
| c_25_ | Between weaker and average | Between weaker and stronger | At least average | At least average | average |
| c_26_ | weaker | Between weak and average | At most average | At least weaker | At most average |
| c_27_ | Between weaker and average | At least weaker | average | At most average | Between weak and average |
| c_28_ | average | strong | At least average | average | Between average and strong |
| c_29_ | Between weaker and average | Between average and strong | Between average and stronger | average | average |
| c_30_ | At least weaker | At most average | average | weak | weaker |
| c_31_ | At most stronger | At least weaker | Between weak and weaker | weak | At most average |
| c_32_ | Between average and strong | Between weaker and stronger | At least average | At least average | At least average |
| c_33_ | At least average | stronger | average | Between average and stronger | average |
| c_34_ | Between average and stronger | At most stronger | Between average and strong | stronger | stronger |
| c_35_ | average | Between average and strong | average | At least weaker | average |
| c_36_ | Between weak and average | Between weaker and stronger | At most stronger | Between weak and weaker | average |
| c_37_ | Between weaker and stronger | At most average | Between average and strong | average | stronger |
| c_38_ | Between weaker and average | average | Between average and stronger | At least weaker | At least weaker |
| c_39_ | At most average | average | Between average and stronger | At most average | At most stronger |
| c_40_ | stronger | At most stronger | Between weaker and stronger | Between average and strong | At least average |
| c_41_ | average | At most average | average | weaker | Between average and strong |
| c_42_ | At most average | Between average and stronger | Between weak and average | stronger | At least weaker |
| c_43_ | At most average | At least average | stronger | average | Between average and stronger |
| c_44_ | average | At least average | average | At most stronger | At most average |
| c_45_ | At least weaker | At most stronger | Between weaker and average | At most average | stronger |
| c_46_ | At most average | At most stronger | Between weaker and stronger | Between weaker and average | At most average |
| c_47_ | At least weaker | Between average and stronger | stronger | average | Between average and strong |
| c_48_ | average | At most average | average | Between average and stronger | stronger |
| c_49_ | Between weaker and average | average | Between weaker and stronger | Between average and strong | Between weaker and average |
| c_50_ | average | At most average | Between average and stronger | At most average | At most stronger |
| c_51_ | Between average and stronger | At most stronger | At least weaker | At least weaker | Between average and strong |
| c_52_ | weaker | average | At most average | Between average and stronger | At most stronger |
| c_53_ | average | Between average and strong | Between weak and average | Between weak and weaker | Between weak and weaker |
